# Circulating plasma microRNA expression profiles associated with virological failure and HIV drug resistance among adults receiving second-line protease inhibitor-based antiretroviral therapy in Zimbabwe

**DOI:** 10.21203/rs.3.rs-10551676/v1

**Published:** 2026-08-07

**Authors:** Justin Mayini, Tendai Washaya, Justen Manasa, Edward Zumbika, Shungu Munyati, Ryman Shoko, Vinie Kouamou, Christine Dahlke

**Affiliations:** Biomedical Research and Training Institute; Biomedical Research and Training Institute; Biomedical Research and Training Institute; National University of Science and Technology; Biomedical Research and Training Institute; University of Lusaka; Infectious Diseases Research Laboratory; Institute for Infection Research and Vaccine Development

**Keywords:** HIV-1, circulating plasma microRNA, virological failure, HIV drug resistance, second-line antiretroviral therapy, Zimbabwe

## Abstract

**Background:**

Virological failure (VF) and HIV drug resistance (HIVDR) remain major challenges to antiretroviral therapy (ART). Plasma viral load (VL) cannot distinguish adherence-related failure from HIVDR, while genotypic resistance testing (GRT) is limited by cost. Circulating plasma microRNAs (miRNAs) may provide complementary host-response markers for monitoring HIV treatment outcomes. We evaluated the expression and discriminatory performance of three miRNAs (hsa-miR-146a-5p, hsa-miR-150–5p, hsa-miR-29a-3p) among adults on protease inhibitor-based second-line ART in Zimbabwe.

**Methods:**

We conducted a multicentre cross-sectional study (January 2023–August 2025) among adults receiving second-line ART for ≥ 12 months. Participants were classified as virologically suppressed (VS; VL < 1 000 copies/mL) or experiencing VF (VL ≥ 1 000 copies/mL). Individuals with VF underwent GRT and were classified as VF without resistance (VF-NR) or with confirmed resistance (VF-R). Plasma miRNA expression was quantified using reverse transcription quantitative PCR. Discriminatory performance was assessed using receiver operating characteristic analysis with bootstrap validation, and independent associations using multivariable logistic regression.

**Results:**

Among 119 participants (40 VS, 33 VF-NR and 46 VF-R), miR-146a-5p expression increased, whereas miR-150–5p and miR-29a-3p declined progressively from VS to VF-R. The combined panel discriminated VF (AUC = 0.893) better than individual biomarkers (ΔAUC = 0.125, P < 0.001) and showed moderate discrimination of HIVDR (AUC = 0.769). Higher miR-146a-5p expression was associated with increased odds of VF and HIVDR, while higher miR-150–5p expression was associated with reduced odds of both outcomes.

**Conclusions:**

A circulating plasma miRNA panel showed promising discrimination of VF and moderate discrimination of HIVDR. These findings support further evaluation of host-response miRNA signatures as complementary tools for VF stratification and prioritisation of confirmatory HIVDR testing.

## Background

Despite the scale-up of antiretroviral therapy (ART), HIV drug resistance (HIVDR) continues to threaten long-term treatment success, particularly in resource-limited settings (RLS)^1^. Sub-Saharan Africa (SSA) carries the largest share of the global HIV burden, where sustained treatment effectiveness remains critical to achieving long-term viral suppression^2^. Although treatment guidelines have increasingly adopted dolutegravir (DTG)-based regimens, many people living with HIV (PLWH) in SSA remain on ritonavir-boosted protease inhibitor (PI)-based second-line ART after switching from failing first-line regimens under earlier World Health Organization (WHO) recommendations^3^. Consequently, these regimens continue to be widely represented in established treatment cohorts in SSA^4^. Although PI-based second-line regimens have a relatively high genetic barrier to resistance, prolonged viraemia during treatment may permit the accumulation of drug resistance mutations (DRMs), potentially compromising future treatment options^5^.

Current HIV treatment monitoring strategies are limited in distinguishing the underlying mechanisms of virological failure (VF). Plasma viral load (VL) testing reliably identifies VF but cannot determine whether persistent viraemia is associated with HIVDR or other causes of treatment failure, like poor adherence. Genotypic resistance testing (GRT) provides direct evidence of HIVDR but remains technically demanding, costly and is not routinely accessible in many RLS. This diagnostic gap has driven interest in complementary, minimally invasive host-response markers that may help characterise the biological heterogeneity of VF and identify individuals who could benefit from confirmatory HIVDR testing.

MicroRNAs (miRNAs) are small non-coding RNAs that regulate gene expression across diverse cellular processes and are stably detectable in plasma^6,7^, making them attractive candidate biomarkers. Altered circulating miRNA profiles have been associated with HIV disease progression, immune activation and treatment response^8–10^. These observations suggest that host miRNA expression may also reflect virological and resistance-associated disease states. However, evidence linking circulating miRNA expression specifically to VF and genotypically confirmed HIVDR remains limited; to our knowledge, no published study has evaluated this among adults receiving second-line PI-based ART in Zimbabwe.

We hypothesised that circulating plasma miRNA expression profiles differ according to virological and HIVDR status and that selected miRNAs would demonstrate discriminatory potential for distinguishing VF from virological suppression (VS), and VF with resistance (VF-R) from VF without detectable resistance (VF-NR). Building on our previous characterisation of HIVDR in this population^11^, this study quantified circulating plasma hsa-miR-29a-3p, hsa-miR-146a-5p and hsa-miR-150–5p expression among adults with VS, VF-NR and VF-R, and evaluated their individual and combined diagnostic performance, and clinical and resistance-related associations, as candidate host-response biomarkers.

## Methods

### Study design and setting

This multicentre cross-sectional study was conducted between January 2023 and August 2025 among adult PLWH receiving second-line PI-based ART in Zimbabwe. Participants were recruited from two tertiary HIV referral centres: the Mpilo Centre of Excellence (MCoE) in Bulawayo and Sally Mugabe Central Hospital (SMCH) in Harare, both of which provide specialised HIV care to patients referred from multiple districts and provinces. Laboratory analyses were performed at the Biomedical Research and Training Institute (BRTI), Harare. The HIVDR characteristics of the study population have been reported previously^11^. The present study represents a separate investigation evaluating three literature-derived circulating plasma miRNAs as candidate host-response markers for stratifying VS, VF and genotypically confirmed HIVDR.

### Study participants, recruitment and specimen collection

Adult PLWH receiving second-line PI-based ART were recruited consecutively at the two study sites to minimise selection bias. Written informed consent was obtained from all participants before enrolment and sample collection. Eligible participants were aged ≥ 18 years, had confirmed HIV-1 infection, had received second-line PI-based ART continuously for at least 12 months, and had VL, treatment regimen and treatment duration data available. Individuals who were pregnant, had documented acute illness or opportunistic co-infection (including active tuberculosis or tuberculosis treatment), or had incomplete clinical or laboratory data were excluded.

An HIV-negative healthy control (HC) group was recruited from the same clinical sites for exploratory descriptive comparison following confirmation of HIV-negative status using the national rapid HIV testing algorithm. However, detectability varied across the three target miRNAs, with a substantial proportion of HC samples yielding undetermined or above-threshold quantification cycle (Cq) values for one or more targets. Consequently, HCs were excluded from the primary inferential analyses and are presented descriptively only, restricting the primary analytical population to adult PLWH receiving second-line PI-based ART.

Participants were classified according to virological and HIVDR status. VS was defined as plasma VL < 1,000 copies/mL, whereas VF was defined as two consecutive plasma VL measurements ≥ 1,000 copies/mL performed at least six months apart, in accordance with WHO recommendations. For participants classified as having VF, the most recent VL measurement was used for subsequent analyses, including descriptive analyses, correlation analyses and regression models. In the final analytical sample, all participants classified as VS had VL reported as target not detected (TND) or < 40 copies/mL. Participants with VF subsequently underwent HIVDR genotyping and were classified as VF without resistance (VF-NR) or VF with resistance (VF-R) according to the Stanford HIV Drug Resistance Database (HIVdb version 9.8)^12^. Clinical group assignment was performed independently of miRNA expression results.

Sample size was estimated for the primary comparison between participants with VF and VS using a standardised effect size approach for independent two-group comparisons^13^. Assuming a moderate-to-large effect size (Cohen’s *d* = 0.65), a two-sided α of 0.05 and 80% power, a minimum of 38 participants per group was required for the primary VF versus VS comparison. To buffer against potential exclusions and incomplete data, the target was rounded up to 40 participants per group. Assuming an HIVDR prevalence of approximately 55% among participants with VF^1,14^, recruitment was increased to approximately 85 participants with VF (≈ 38 VF-NR and ≈ 47 VF-R), giving an overall recruitment target of approximately 125 PLWH. HCs were recruited pragmatically and were not included in the formal sample size calculation.

At enrolment, 5 mL of venous whole blood was collected into EDTA tubes and processed within two hours of phlebotomy. Plasma was separated by centrifugation (1,500 × *g*, 10 min) and aliquoted into three 1 mL fractions. Plasma samples exhibiting visible haemolysis (pink to red discolouration on visual inspection) were excluded before RNA extraction because haemolysis may alter circulating miRNA profiles; formal haemolysis assessment using spectrophotometric or miRNA-based methods was not performed. One aliquot was used for HIV-1 VL quantification at the collection site using the Xpert HIV-1 Viral Load assay on the GeneXpert Dx System (Cepheid, Sunnyvale, CA, USA)^15^, while the remaining aliquots, designated for HIVDR genotyping (where applicable) and plasma miRNA analysis, were stored at − 20°C, transported on ice to the BRTI, and subsequently stored at − 80°C until laboratory analysis. For quantitative analyses, VL results reported as TND were assigned a value of 1.0 log_10_ copies/mL, whereas detectable results below the lower limit of quantification (< 40 copies/mL) were assigned a value of 1.6 log_10_ copies/mL. Demographic and clinical data, including age, sex, ART regimen, ART duration, PI duration and VL results, were extracted from hospital records using a standardised data collection tool.

### HIV drug resistance genotyping and resistance interpretation

HIVDR genotyping was performed for participants with VF by Sanger sequencing of the HIV-1 *pol* gene, encompassing the *protease* (PR) and *reverse transcriptase* (RT) coding regions, as previously described^11^. Consensus sequences were analysed using the Stanford University HIVdb to identify DRMs and predict antiretroviral (ARV) susceptibility. GenBank accession numbers for all sequences included in the analyses are provided in **Supplementary Table S1**.

Clinically relevant HIVDR was defined as the presence of one or more DRMs conferring a cumulative Stanford University HIVdb penalty score of ≥ 15, corresponding to at least low-level resistance. Participants were subsequently classified as VF-R or VF-NR based on these resistance interpretation criteria. Secondary resistance characteristics evaluated included the presence of major PI DRMs, PI mutation burden, nucleoside reverse transcriptase inhibitor (NRTI) and non-nucleoside reverse transcriptase inhibitor (NNRTI) mutation counts.

### Selection of candidate miRNAs

Candidate miRNAs hsa-miR-29a-3p, hsa-miR-146a-5p and hsa-miR-150–5p were selected *a priori* following a scoping review of published studies investigating circulating plasma miRNAs in HIV infection. Selection was informed by their reported roles in HIV-1 pathogenesis and host–virus interactions and by previous associations with immune regulation, ART response and HIV disease progression^8,10,16^. The specific mature miRNA strands were selected based on the forms reported in the relevant plasma and HIV literature. miRNA nomenclature followed miRBase version 22^17^.

### RNA extraction, reverse transcription and RT-qPCR

Total RNA, including small RNAs, was extracted from 200 μL plasma using the miRNeasy Serum/Plasma Advanced Kit (Qiagen, Hilden, Germany) according to the manufacturer’s instructions. Frozen plasma was thawed on ice and clarified by centrifugation (8,000 × *g*, 5 min) before lysis. The synthetic exogenous spike-in control *cel-miR*-39 (Qiagen) was added to each sample before RNA extraction to monitor extraction efficiency and technical consistency. RNA was eluted in 20 μL RNase-free water, and concentration and purity (A260/A280) were assessed using a DeNovix DS-11 spectrophotometer (DeNovix Inc., Wilmington, DE, USA). Because cell-free plasma RNA yields are typically low and variable, a fixed input volume of 5 μL RNA eluate was used for reverse transcription for all samples.

Complementary DNA (cDNA) was synthesised using TaqMan^™^ Small RNA Assays and MultiScribe^™^ Reverse Transcriptase (Thermo Fisher Scientific, Waltham, MA, USA; assay identifiers are provided in **Supplementary Table S2**) according to the manufacturer’s protocol. Separate reverse transcription reactions were performed for each target miRNA (miR-29a-3p, miR-146a-5p and miR-150–5p), the candidate endogenous reference miRNAs (miR-16–5p, miR-93-5p and miR-484-5p), and the exogenous spike-in control cel-miR-39 using target-specific stem-loop primers. Thermal cycling conditions comprised 16°C for 30 min, 42°C for 30 min and 85°C for 5 min, after which cDNA was stored at − 20°C until quantitative PCR (qPCR).

RT-qPCR was performed using TaqMan^™^ Small RNA Assays with TaqMan^™^ Fast Advanced Master Mix on a QuantStudio^™^ 3 Real-Time PCR System (Thermo Fisher Scientific) in 10 μL reaction volumes following the Minimum Information for Publication of Quantitative Real-Time PCR Experiments (MIQE) guidelines^18^. Amplification consisted of an initial denaturation at 95°C for 20 s followed by 40 cycles of 95°C for 1 s and 60°C for 20 s. All reactions were performed in duplicate with no-template controls (NTCs) included in each run and selected samples were included as inter-plate calibrators (IPCs) to monitor run-to-run consistency. Duplicate Cq values differing by > 0.5 cycles were repeated where sufficient RNA was available; otherwise, the measurement was excluded from downstream analysis.

Cq values > 35 or undetermined amplification were considered below the predefined quantification threshold and were excluded from subsequent expression analyses.

#### Reference miRNA validation and expression normalisation

Three candidate endogenous reference miRNAs (miR-16–5p, miR-93-5p and miR-484-5p) were evaluated for expression stability across the VS, VF-NR and VF-R groups using the standard deviation (SD) and coefficient of variation (CV) of Cq values. miR-93-5p and miR-484-5p demonstrated the greatest expression stability and were therefore selected as endogenous reference miRNAs, whereas miR-16–5p showed greater variability and was excluded from the normalisation strategy (**Supplementary Table S3**).

Target miRNA expression was normalised using the geometric mean of miR-93-5p and miR-484-5p, calculated from the arithmetic mean of their Cq values, consistent with recommendations supporting the use of multiple endogenous reference genes for RT-qPCR analyses^19^. Normalised expression was calculated as:

$$\Delta Cq=Cq_{\text{target}}-\frac{Cq_{\text{miR-93-5p}}+Cq_{\text{miR-484-5p}}}{2}$$

The exogenous spike-in control cel-miR-39 was used to monitor RNA extraction efficiency and technical consistency but was not used for expression normalisation.

Relative fold change (FC) was calculated using the 2^−ΔΔCq^ method described by Livak and Schmittgen^20^. ΔΔCq values were calculated relative to the mean ΔCq of the VS group (calibrator). Because FC provides a descriptive measure of relative expression rather than a statistical test, conventional mean-based 2^−ΔΔCq^ calculations were retained irrespective of the distribution of individual observations. Statistical comparisons were performed using non-parametric analyses of ΔCq values. As a sensitivity analysis, FC estimates calculated from group median ΔCq values are presented alongside the conventional mean-based estimates.

Lower ΔCq values indicate higher target miRNA expression. For graphical presentation, correlation analyses and regression modelling, ΔCq values were transformed to − ΔCq so that higher values corresponded to higher relative miRNA expression.

### Statistical analysis

Continuous variables were summarised using medians and interquartile ranges (IQRs), and categorical variables as frequencies and percentages. Normality was assessed using the Shapiro–Wilk test and visual inspection of data distributions. Because miRNA expression data were non-normally distributed, non-parametric methods were used throughout. Statistical significance was defined as a two-sided *P* < 0.05 unless otherwise specified. Missing data were handled using complete-case analysis, such that participants were excluded only from analyses involving the specific missing variable(s). Statistical analyses were performed using Stata version 17 (StataCorp LLC, College Station, TX, USA)^21^.

#### Group comparisons

Differences in normalised miRNA expression (ΔCq) between two groups were assessed using the Mann–Whitney *U* test, while comparisons across the three clinical groups (VS, VF-NR and VF-R) were performed using the Kruskal–Wallis test. Significant Kruskal–Wallis results were followed by pairwise Mann–Whitney *U* tests with Holm adjustment for multiple comparisons. Effect sizes were calculated as *r* = *Z*/√*N* and interpreted as small (0.10–0.29), moderate (0.30–0.49) or large (≥ 0.50). Because the study evaluated three pre-specified candidate circulating plasma miRNAs selected *a priori*, no adjustment for multiple testing was applied across the individual markers.

#### Correlation analysis;

Associations between circulating plasma miRNA expression (−ΔCq) and clinical or molecular variables, including VL, ART duration, PI duration, NRTI mutation count, NNRTI mutation count, and major PI mutation count, were assessed using Spearman’s rank correlation coefficient (ρ). Correlations with VL were evaluated in both the overall study population and, as a pre-specified subgroup analysis, among participants with VF. To control the false discovery rate (FDR), the Benjamini–Hochberg procedure was applied across all reported correlation analyses, and adjusted q-values are reported. Scatter plots with fitted linear trend lines were generated to illustrate significant associations; statistical inference was based exclusively on Spearman correlation coefficients.

#### Diagnostic performance

Receiver operating characteristic (ROC) curve analysis was used to evaluate the discriminatory performance of individual circulating plasma miRNAs and combined models for distinguishing VF from VS and VF-R from VF-NR. For individual miRNAs, ROC curves were generated using − ΔCq values, whereas combined models were constructed using multivariable logistic regression, with predicted probabilities used to generate ROC curves. Diagnostic performance was summarised using the area under the ROC curve (AUC) with 95% confidence intervals (CIs). Optimal thresholds were identified using the Youden index. Sensitivity, specificity, positive predictive value (PPV) and negative predictive value (NPV) were reported at the selected thresholds, with PPV/NPV additionally estimated across assumed VF prevalences of 20%, 25% and 30% to illustrate performance across clinically plausible settings. In the absence of an independent external validation dataset, model performance was internally validated using 2,000 bootstrap resamples. Model optimism was estimated as the mean difference between bootstrap-sample and original-sample performance, and optimism-corrected AUCs were calculated by subtracting the estimated optimism from the apparent AUC.

#### Regression analysis

Binary logistic regression was used to evaluate independent associations between circulating plasma miRNA expression (−ΔCq) and VF (VF versus VS) and HIVDR (VF-R versus VF-NR). Models were adjusted for age, sex, ART duration and PI duration. Results were presented as adjusted odds ratios (aORs) with 95% confidence intervals (CIs). Model fit was assessed using the Hosmer–Lemeshow goodness-of-fit test^22^, while model discrimination was evaluated using the AUC.

## Results

### Participant flow and baseline characteristics

**Figure 1:**
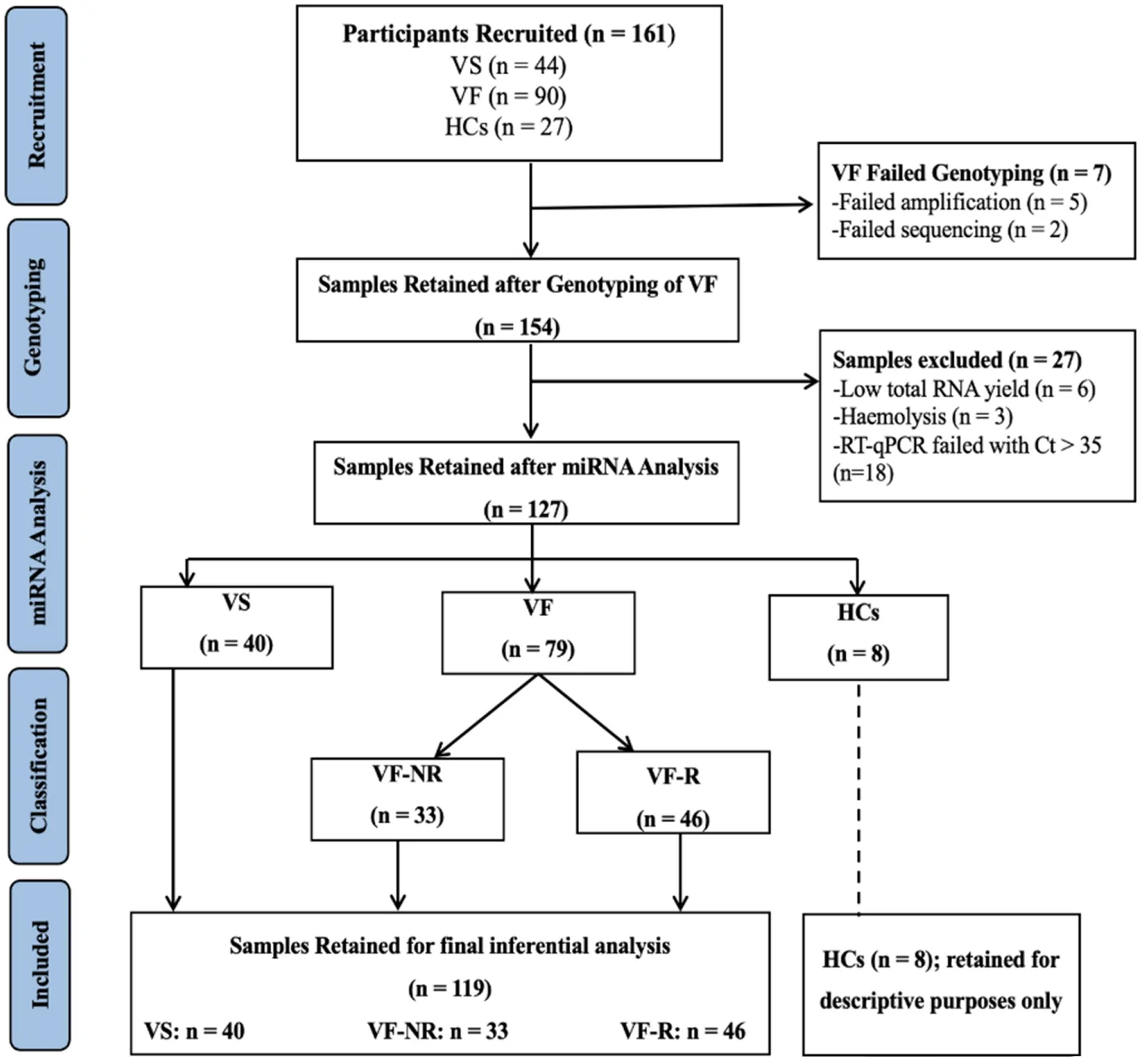
Study flow diagram illustrating participant enrolment, HIV drug resistance genotyping, miRNA workflow exclusions and final clinical group classification. HCs, healthy controls; HIVDR, HIV drug resistance; GRT,genotypic resistance testing; miRNA, microRNA; PLWH, people living with HIV; RT-qPCR, reverse transcription quantitative polymerase chain reaction; VF, virological failure; VF-NR, virological failure without resistance; VF-R,virological failure with resistance; VS, virological suppression.

A total of 161 participants were enrolled, comprising 90 with VF, 44 with VS and 27 HIV-negative healthy controls (HCs). Among participants with VF, seven were excluded following unsuccessful HIVDR genotyping due to amplification (n = 5) or sequencing failure (n = 2), leaving 154 samples eligible for miRNA analysis. A further 27 samples were excluded during the miRNA workflow because of haemolysis (n = 3), low RNA yield (n = 6) or RT-qPCR quality-control failure (Cq > 35; n = 18). The final analytical study sample comprised 127 participants, including 119 adult PLWH and eight HCs with quantifiable expression across all three target miRNAs. Among PLWH, 40 were classified as VS, 33 as VF without resistance (VF-NR) and 46 as VF with confirmed resistance (VF-R). Owing to the limited number of HCs with quantifiable expression across all three target miRNAs, HCs were retained for exploratory descriptive analyses only and excluded from inferential statistical comparisons (Fig. 1). Among the 27 HCs recruited, successful amplification was observed in 15/27 (55.6%) samples for miR-29a-3p, 16/27 (59.3%) for miR-146a-5p and 18/27 (66.7%) for miR-150–5p. Descriptive expression data for HCs are presented in **Supplementary Table 4**.

RNA concentrations ranged from 2.76 to 21.80 ng/μL, and A260/A280 ratios ranged from 1.70 to 2.33. The exogenous spike-in control (*cel-miR*-39–3p) demonstrated consistent amplification across all samples (median Cq 24.4, IQR 23.9–25.2).

Baseline demographic and clinical characteristics of the final analytical sample are summarised in Table 1. Age and sex distributions were comparable across the three clinical groups, with no significant differences observed (both *P* > 0.05). In contrast, cumulative ART duration differed significantly, increasing progressively from the VS group to the VF-R group (*P* < 0.001), whereas PI duration and ART regimen distribution were similar across groups.

Among participants with VF, plasma VL did not differ between the VF-NR and VF-R groups (*P* = 0.960). However, participants with VF-R harboured significantly greater numbers of major PI, NRTI and NNRTI DRMs than those with VF-NR (all *P* < 0.001). These well-defined clinical groups provided the framework for subsequent comparisons of circulating miRNA expression according to virological and HIVDR status.

#### Differential circulating plasma miRNA expression according to virological and HIV drug resistance status

**Figure 2:**
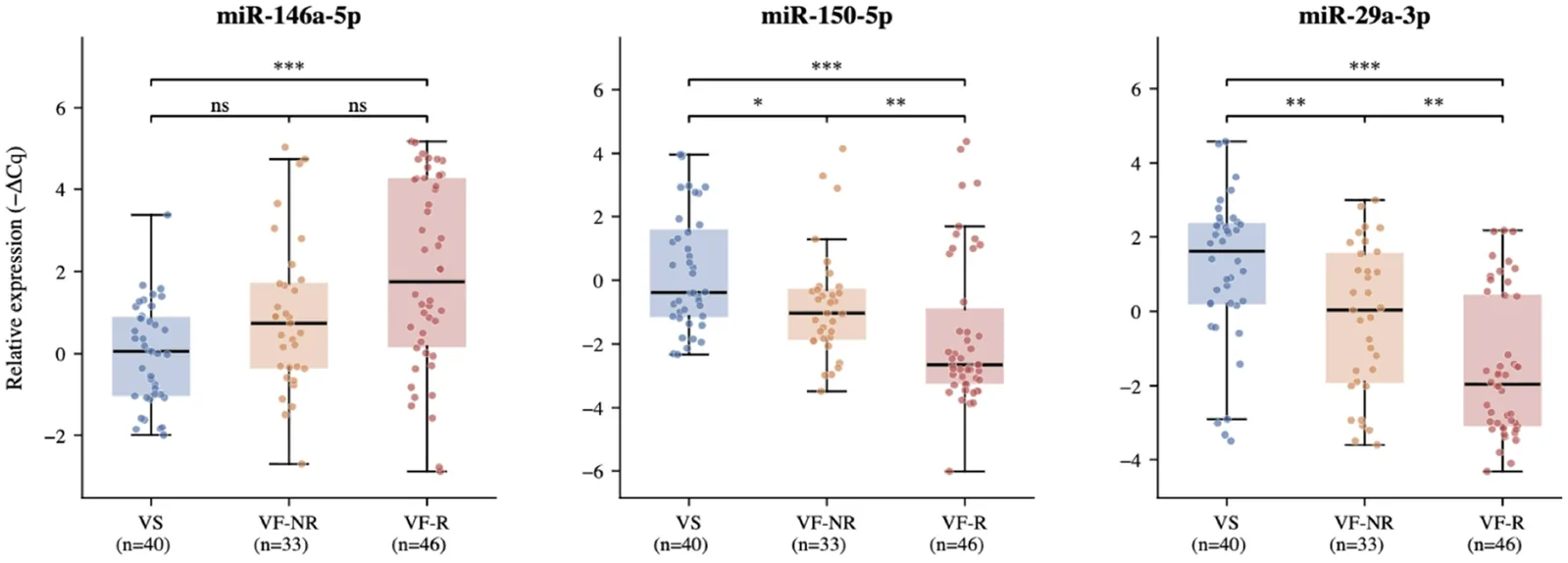
Differential expression of circulating plasma miRNAs according to virological failure and HIV drug resistance status. Boxplots show the median (horizontal line), interquartile range (box), and range excluding outliers (whiskers) of circulating plasma miRNA expression (−ΔCq) across the three clinical groups, with individual participant values overlaid. Higher −ΔCq values indicate higher relative miRNA expression. Overall group differences were assessed using the Kruskal–Wallis test, followed by Holm-adjusted pairwise Mann–Whitney U tests. Statistical significance is indicated as * (*P* < 0.050), ** (*P* < 0.010), *** (*P*< 0.001), and ns (not significant; Holm-adjusted). VS, virological suppression; VF-NR, virological failure without drug resistance; VF-R, virological failure with drug resistance.

Relative expression of all three candidate miRNAs differed significantly across the three clinical groups (miR-146a-5p: *H* = 16.61, *P* < 0.001; miR-150–5p: *H* = 24.37, *P* < 0.001; miR-29a-3p: *H* = 28.86, *P* < 0.001; Table 2). As illustrated in Figure 2, miR-146a-5p exhibited progressively lower ΔCq values (corresponding to higher relative expression) from VS to VF-R, whereas miR-150–5p and miR-29a-3p exhibited progressively higher ΔCq values (corresponding to lower relative expression) across the same clinical groups. Relative FC estimates derived using the conventional 2^−ΔΔCq^ method supported these findings. Compared with the VS group, **mean-based** FC estimates showed that miR-146a-5p expression increased approximately 1.9-fold in VF-NR and 3.8-fold in VF-R, whereas miR-150–5p and miR-29a-3p were progressively downregulated, declining to 0.47- and 0.39-fold of VS levels in VF-NR and to 0.25- and 0.16-fold in VF-R, respectively (Table 2). Median-based FC estimates showed the same direction of expression change for all three miRNAs, although the magnitude of change differed modestly, particularly for miR-29a-3p in the VF-R group (median-based FC = 0.08 versus mean-based FC = 0.16). Overall, the sensitivity analysis confirmed that the direction and interpretation of differential expression were robust to the choice of summary statistic used for fold-change estimation.

Visual inspection of the expression distributions (Figure 2), together with Holm-adjusted post-hoc analyses (Table 3), identified two distinct expression patterns among the candidate miRNAs. MiR-150–5p and miR-29a-3p exhibited a progressive reduction in relative expression across the clinical continuum from VS to VF-NR and finally to VF-R. All three pairwise comparisons remained statistically significant after Holm correction (all adjusted *p* ≤ 0.020), with small-to-moderate effect sizes for comparisons between adjacent clinical groups (*r* = 0.272–0.338) and moderate-to-large effect sizes between VS and VF-R (*r* = 0.495–0.551), supporting a progressive association with worsening VF and HIVDR.

In contrast, miR-146a-5p displayed a distinct expression profile. Although overall expression differed significantly among the three clinical groups, only the comparison between VS and VF-R remained significant following Holm adjustment (adjusted *P* < 0.001; *r* = 0.426). Comparisons between VS and VF-NR and between VF-NR and VF-R were no longer statistically significant after adjustment (both adjusted *P* = 0.071). These findings indicate that miR-146a-5p primarily discriminated between VS individuals and those with VF-R, rather than between adjacent stages of disease progression.

### Associations between circulating plasma miRNA expression and clinical, virological and HIV drug resistance characteristics

Across the overall study population, expression of all three candidate miRNAs was significantly associated with plasma VL after correction for multiple testing (Table 4). MiR-29a-3p and miR-150–5p exhibited inverse correlations with VL (ρ = −0.365, q < 0.001 and ρ = −0.322, q < 0.001, respectively), whereas miR-146a-5p showed a positive correlation (ρ = 0.252, q = 0.025). In the prespecified subgroup analysis restricted to participants with VF, none of the three miRNAs was significantly correlated with VL after correction for multiple testing (all |ρ| < 0.06, q ≥ 0.752). Relative expression of miR-29a-3p and miR-150–5p also showed significant inverse correlations with ART duration (ρ = −0.359, q < 0.001 and ρ = −0.227, q = 0.048, respectively), whereas miR-146a-5p was not significantly associated with ART duration (q = 0.355). No significant associations were observed between PI duration and expression of any of the three miRNAs after correction for multiple testing.

Within the VF subgroup, miR-146a-5p was the only miRNA significantly associated with HIVDR characteristics after FDR correction. Specifically, miR-146a-5p expression correlated positively with both NRTI mutation count (ρ = 0.354, q = 0.005) and major PI mutation count (ρ = 0.281, q = 0.042) (Table 4; **Supplementary Figure 1**). Neither miR-29a-3p nor miR-150–5p showed significant associations with mutation counts after multiple-testing correction. Correlations with NNRTI mutation count observed before adjustment did not remain statistically significant following Benjamini–Hochberg correction (q = 0.107–0.122) (Table 4). Complete Spearman correlation coefficients and corresponding raw P values are provided in **Supplementary Table 5**.

### Diagnostic performance of individual and combined miRNA models

**Figure 3:**
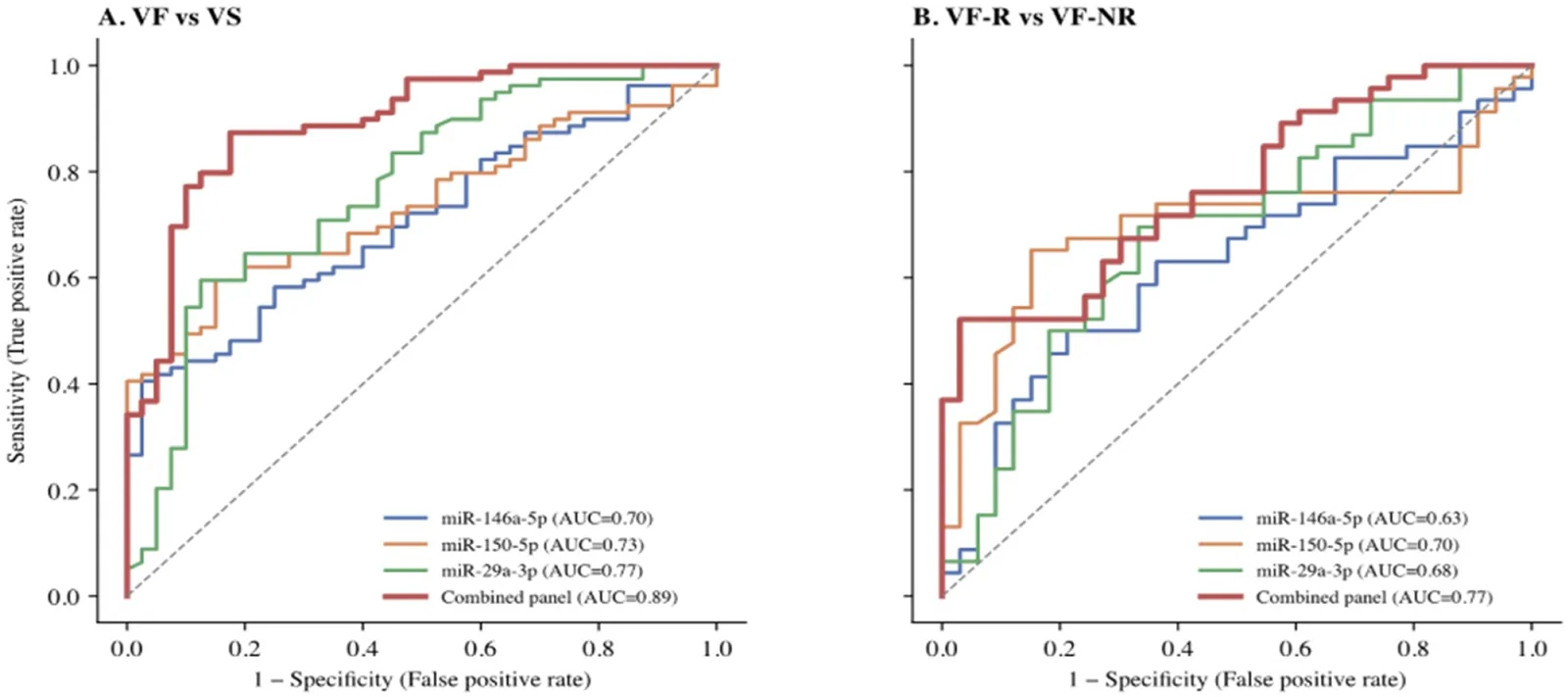
Receiver operating characteristic (ROC) curves for individual and combined circulating plasma miRNA models. **(A)** ROC curves for discrimination between participants with virological failure (VF) and virological suppression (VS). **(B)** ROC curves for discrimination between virological failure with drug resistance (VF-R) and virological failure without resistance (VF-NR). The combined panel was derived from a logistic regression model incorporating miR-146a-5p, miR-150–5p and miR-29a-3p. The diagonal dashed line represents discrimination expected by chance (AUC = 0.50). Diagnostic performance metrics and bootstrap-derived 95% confidence intervals are presented in Table 5.

The diagnostic performance of each biomarker and the combined panel is summarised in Table 5, with ROC curves presented in Figure 3. For discrimination of participants with **VF versus VS,** individual miRNAs showed fair diagnostic performance, with AUCs ranging from **0.704** for miR-146a-5p to **0.768** for miR-29a-3p (Table 5). The combined three-miRNA logistic regression model demonstrated the highest discriminatory performance, with an apparent AUC of **0.893** (95% CI: 0.829–0.949), **87.3% sensitivity**, and **82.5% specificity** at the Youden-optimal threshold. The optimism-corrected AUC following internal validation was 0.883, indicating minimal evidence of model overfitting. Paired bootstrap analysis demonstrated that the combined panel significantly improved discrimination compared with the best-performing individual biomarker, miR-29a-3p (ΔAUC = **0.125**, 95% CI: 0.061–0.218; *P* < 0.001; Table 6).

For discrimination of participants with **VF-R versus VF-NR**, diagnostic performance was more modest (Table 5). Among the individual biomarkers, miR-150–5p achieved the highest AUC (**0.699),** followed by miR-29a-3p (**0.683**), while miR-146a-5p exhibited the lowest discriminatory performance (AUC **0.631**, 95% CI: 0.499–0.750). The combined three-miRNA model achieved an apparent AUC of **0.769** (95% CI: 0.660–0.862), with **52.2% sensitivity** and **97.0% specificity**, and an optimism-corrected AUC of **0.740**. Although the combined panel achieved a higher AUC than the best-performing individual biomarker, the paired bootstrap comparison showed that this improvement was not statistically significant (ΔAUC = **0.070**, 95% CI: −0.015 to 0.259; *P* = 0.109; Table 6).

For the VF versus VS comparison, PPV and NPV were estimated for assumed VF prevalences of 20%, 25% and 30% (**Supplementary Table 6**). At an assumed prevalence of 25%, the combined three-miRNA panel yielded an estimated PPV of **62.5%** and NPV of **95.1%**. Across the evaluated prevalence range, PPV increased from **55.5%** to **68.1%**, whereas NPV remained consistently high, ranging from **93.8%** to **96.3%**.

### Multivariable logistic regression analysis

The multivariable logistic regression analyses to determine whether circulating plasma miRNA expression was independently associated with VF and HIVDR after adjustment for age, sex, ART duration and PI duration are presented in Table 7. For the comparison of **VF versus VS,** higher expression of **miR-146a-5p** was independently associated with higher odds of VF (aOR = **3.11**, 95% CI: 1.80–5.39; *P* < 0.001). Conversely, higher expression of **miR-150–5p** was associated with significantly lower odds of VF (aOR = **0.43**, 95% CI: 0.26–0.70; *P* = 0.001). **MiR-29a-3p** was not independently associated with VF after adjustment for the other covariates (aOR = 0.77, 95% CI: 0.56–1.06; *P* = 0.110). Among the clinical variables, longer ART duration remained independently associated with increased odds of VF (aOR = **1.32** per year, 95% CI: 1.09–1.60; *P* = 0.005), whereas age, sex and PI duration were not significantly associated with the outcome.

Among participants with VF, higher expression of **miR-146a-5p** was also independently associated with HIVDR (aOR = **1.44,** 95% CI: 1.04–1.99; *P* = 0.027). In contrast, higher expression of **miR-150–5p** remained independently associated with lower odds of HIVDR (aOR = **0.67**, 95% CI: 0.46–0.96; *P* = 0.031). **MiR-29a-3p** was not independently associated with HIVDR after adjustment (aOR = 1.12, 95% CI: 0.81–1.55; *P* = 0.490). Longer ART duration also remained independently associated with HIVDR (aOR = **1.18** per year, 95% CI: 1.01–1.38; *P* = 0.040), while age, sex and PI duration were not significantly associated with the outcome.

## Discussion

### Principal findings

A principal finding of this study was that three literature-derived candidate circulating plasma miRNAs exhibited distinct expression patterns across VS, VF-NR, and VF-R among adult PLWH receiving second-line PI-based ART in Zimbabwe. Circulating plasma miR-146a-5p expression increased progressively from VS to VF-NR and VF-R, whereas miR-150–5p and miR-29a-3p demonstrated progressive reductions across the same clinical continuum. Notably, this discrimination was not uniform across the three candidates: miR-150–5p and miR-29a-3p differed significantly between every pairwise comparison after correction for multiple testing, indicating a consistent stepwise relationship with worsening treatment outcomes, whereas miR-146a-5p differed significantly only between VS and VF-R, with differences between adjacent stages no longer significant after correction. This suggests miR-146a-5p more specifically marks established, advanced treatment failure rather than tracking a continuous disease gradient. Among the individual miRNAs, miR-29a-3p demonstrated the highest discriminatory performance for identifying VF, while the combined three-miRNA panel achieved the greatest overall diagnostic accuracy, though discrimination of HIVDR specifically was more modest, consistent with a stratification rather than diagnostic role.

These findings extend previous reports of altered expression of miR-146a-5p^10,16,23^, miR-150–5p^10,23^, and miR-29a-3p^24–28^ *expression during HIV infection, showing that expression also varies by VS and HIVDR status in a Southern African cohort with genotypically confirmed resistance*. Unlike previous studies that primarily examined associations with VL, immune status or treatment response^8,9^ the present study evaluated these candidate miRNAs across clinically relevant treatment outcomes using virological, genotypic and multivariable analytical approaches. Collectively, the findings support the potential utility of circulating plasma host-response miRNA signatures for identifying individuals who may benefit from further investigation for treatment failure and confirmatory HIVDR testing, particularly in RLS where access to GRT remains constrained.

### Interpretation of circulating plasma miRNA expression

An important observation was that changes in circulating plasma miRNA expression were already evident among participants with VF in whom no major DRMs were detected (VF-NR). This suggests that host-response alterations accompany VF before, or independently of, the development of genotypically detectable resistance. However, the VF-NR group is likely to represent a clinically heterogeneous population. In the absence of objective adherence measures, such as pharmacy refill records, pill counts or therapeutic drug monitoring, VF in these individuals may have resulted from suboptimal adherence, treatment interruptions, pharmacokinetic variability, recently emerging resistance below the detection threshold of Sanger sequencing^29,30^, or combinations of these mechanisms. Given this heterogeneity, expression differences between VS and VF-NR likely reflect host responses to VF broadly, rather than a single mechanism. Across the overall study population, expression of all three miRNAs correlated significantly with plasma VL; however, these correlations were no longer evident when the analysis was restricted to participants with VF. This indicates that the observed associations chiefly reflect the categorical distinction between VS and failing individuals, rather than a continuous relationship with the magnitude of viraemia once failure has occurred.

Among the three candidate miRNAs, circulating plasma miR-146a-5p demonstrated the clearest progressive increase in expression from VS through VF-NR to VF-R and was the only miRNA that remained significantly associated with resistance mutation burden after correction for multiple testing, correlating with both major PI mutation count and NRTI mutation count, and remaining independently associated with HIVDR in multivariable regression. *This is biologically plausible given miR-146a-5p’s recognised role in NF-κB-dependent regulation of innate immune and inflammatory* signalling^31^, *and prior reports of its induction during active HIV replication and immune* activation^16,23^*, both processes expected to intensify with sustained viraemia and DRM accumulation*. The progressive increase across the three clinical groups further suggests that miR-146a-5p reflects the cumulative effects of ongoing viraemia, immune activation and treatment failure rather than resistance alone.

In contrast, circulating plasma miR-150–5p and miR-29a-3p demonstrated progressively lower expression across worsening treatment outcomes, and neither remained associated with resistance mutation burden after correction for multiple testing. Both miRNAs have recognised roles in maintaining immune homeostasis and regulating host responses to HIV infection^10,23–28^. Reduced expression among participants with VF, particularly the VF-R group, is consistent with increasing immune dysregulation accompanying persistent viraemia. Collectively, these findings indicate that the three circulating plasma miRNAs capture complementary aspects of the host response to VF and HIVDR. Rather than reflecting a single biological pathway, the combined expression profile integrates markers associated with immune activation, inflammatory regulation and antiviral responses. This complementary behaviour probably explains why the combined three-miRNA model consistently demonstrated better discrimination of VF than the individual miRNAs alone, despite only modest improvements for identifying HIVDR specifically.

### Clinical implications

The findings of this study have potential implications for the management of VF in RLS, where access to GRT remains restricted by cost, laboratory infrastructure and turnaround time^3^. Although plasma VL testing effectively identifies VF, it cannot distinguish treatment failure resulting from HIVDR from that caused by other factors, including suboptimal adherence; because circulating plasma miRNAs reflect the host response rather than viral genetic mutations, they may offer biological information that is complementary to, rather than redundant with, conventional GRT. Given the combined panel’s modest sensitivity for HIVDR (52.2%), these host-response signatures are best positioned not as standalone diagnostic tests, but as a means of stratifying individuals with VF according to their likelihood of harbouring resistance-associated biological changes, thereby helping to prioritise patients for confirmatory GRT where testing capacity is limited.

The combined three-miRNA model achieved the greatest overall discrimination for VF (AUC=0.893), significantly outperforming the best individual miRNA. For HIVDR, the panel achieved moderate absolute discrimination (AUC=0.769) and good specificity, but its improvement over the best-performing individual miRNA was modest and not statistically significant (ΔAUC=0.070). This pattern suggests that combining miRNAs reflecting complementary host-response pathways improves discrimination of treatment status more readily than it provides disease-specific signatures of drug resistance, consistent with panel-based improvements over individual markers reported in other disease contexts^32^. Prospective longitudinal studies, external validation in independent populations and standardisation of analytical workflows will be essential before these candidate circulating plasma miRNAs can be considered for routine clinical application.

These findings should also be interpreted in the context of the ongoing global transition to DTG-based ART. Although ritonavir-boosted PI-based second-line regimens remain in widespread use among established treatment populations in SSA^4^, increasing numbers of patients are receiving DTG-based regimens, which have a distinct resistance profile and a comparatively high genetic barrier to resistance. Because the candidate miRNAs evaluated in this study reflect host immune and inflammatory responses associated with VF rather than PI-specific pharmacological effects, it is biologically plausible that similar host-response signatures may extend to DTG-treated populations. However, this hypothesis requires direct empirical validation, as the biological drivers, kinetics and resistance mechanisms underlying VF on integrase inhibitor-based regimens may differ substantially from those observed with PI-based therapy. Validation of these circulating plasma miRNA signatures in DTG-treated populations therefore represents an important priority for future research, particularly as DTG-based regimens have become the recommended first- and second-line standard of care in most high HIV-burden settings.

### Strengths and limitations

This study has several important strengths. First, it provides one of the first evaluations of literature-derived circulating plasma miRNA candidates for stratifying VS, VF and HIVDR among adults receiving second-line PI-based ART in Zimbabwe, addressing an important evidence gap from SSA. Second, HIVDR was defined using GRT interpreted with the Stanford University HIVdb, providing a robust reference standard for resistance classification. Third, rigorous laboratory quality assurance procedures, including optimisation of RT-qPCR conditions, validated endogenous reference miRNAs for expression normalisation^19,33^, duplicate amplification and predefined quality-control criteria^18^, enhanced the reliability of the circulating plasma miRNA measurements. Fourth, the study incorporated a comprehensive analytical framework that included multivariable regression, ROC analyses and bootstrap internal validation. Finally, adjustment for relevant clinical characteristics strengthened the interpretation of the observed associations between circulating plasma miRNA expression and treatment outcomes.

Several limitations should be considered when interpreting these findings. First, the cross-sectional design precludes assessment of temporal relationships or determination of whether changes in circulating plasma miRNA expression precede or follow the development of VF and HIVDR. Second, although the overall sample size was adequate for the primary analyses, the number of participants with genotypically confirmed HIVDR remained relatively modest, which may have limited statistical power for some subgroup comparisons. Third, only three candidate circulating plasma miRNAs were evaluated, and other host-response signatures may provide additional discriminatory value. Fourth, objective measures of treatment adherence, such as pharmacy refill records, pill counts or therapeutic drug monitoring, were not available, and Cluster of Differentiation 4-positive (CD4+) T-cell counts and other immunological parameters were not assessed; consequently, the VF-NR group probably includes a mixture of true resistance-free failure and cases attributable to suboptimal adherence, pharmacokinetic variability, or emerging resistance below the detection threshold of Sanger sequencing. Fifth, conventional Sanger sequencing cannot reliably detect lowfrequency viral variants, potentially resulting in underestimation of minority resistance mutations^29,30^.

Sixth, haemolysis was assessed by visual inspection rather than by objective spectrophotometric or miRNA-based methods; residual effects of subclinical haemolysis cannot therefore be completely excluded, and given that miR-16–5p is a known erythrocyte-enriched miRNA whose plasma levels and suitability as a reference gene are known to be altered by haemolysis^34^, this represents one possible contributor to the greater intergroup variability observed for miR-16–5p and its subsequent exclusion from the normalisation strategy. However, additional biological and technical factors may also have contributed to this variability. Seventh, because all participants classified as VS had VLs reported as TND or below 40 copies/mL, and all participants with VF had VLs ≥1,000 copies/mL, the analytical population did not include individuals with low-level viraemia (40–999 copies/mL); diagnostic performance estimates may therefore be inflated relative to real-world clinical use, where this ambiguous range is common and clinically important. Finally, most HCs yielded undetermined or late Cq values for one or more target miRNAs and were therefore analysed descriptively only; this may reflect genuinely low circulating abundance of these miRNAs in the absence of HIV-associated immune activation. This explanation is best supported for miR-150–5p specifically, *given evidence that lymphocyte activation drives its extracellular release and serum* elevation^35^*; whether this extends to miR-146a-5p and miR-29a-3p is untested but plausible given their similar roles in inflammatory/antiviral signalling*. Technical factors related to plasma RNA concentration, extraction efficiency or stochastic detection near the assay limit cannot be excluded for any of the three miRNAs, and these explanations could not be distinguished with the data available. Collectively, these factors may have introduced residual confounding and limit the generalisability of the findings beyond adults receiving second-line PI-based ART in Zimbabwe.

## Conclusion

In conclusion, this study demonstrates that three literature-derived candidate circulating plasma miRNAs exhibit distinct expression patterns across VS, VF-NR and VF-R among adults receiving second-line PI-based ART. The progressive increase in circulating plasma miR-146a-5p expression and corresponding reductions in miR-150–5p and miR-29a-3p support the concept that host-response miRNA signatures reflect clinically meaningful differences in treatment status. Although the combined three-miRNA model showed encouraging discriminatory performance for VF, its value for identifying HIVDR appears to lie primarily in supporting host-response stratification and prioritisation for confirmatory GRT rather than replacing existing diagnostic approaches. These findings provide preliminary evidence supporting further evaluation of circulating plasma miRNA signatures as candidate biomarkers for monitoring treatment outcomes. Future longitudinal, multicentre studies incorporating broader miRNA panels, objective adherence measures and external validation will be essential to determine their clinical utility and implementation in HIV care.

## Supplementary Files

This is a list of supplementary files associated with this preprint. Click to download.

- JMayinimiRNASupplementaryMaterial.docx

## Figures and Tables

**Table 1:** Demographic, clinical and virological characteristics of the analytical study sample

Characteristic	VS (n = 40)	VF-NR (n = 33)	VF-R (n = 46)	*P*-value
Age (years), median (IQR)	37.5 (27.0–48.2)	29.0 (25.0–44.0)	39.0 (27.0–48.0)	0.458
Female, n (%)	20 (50.0)	19 (57.6)	24 (52.2)	0.805
ART duration (years), median (IQR)†	8.5 (7.0–12.2)	11.0 (7.5–15.0)	15.0 (11.0–16.0)	**< 0.001**
PI duration (years), median (IQR)	5.0 (3.8–7.0)	4.0 (3.0–7.0)	5.0 (4.0–7.0)	0.598
VL (log_10_ copies/mL), median (IQR)	1.0 (1.0–1.0)	4.80 (4.00–5.20)	4.75 (4.10–5.20)	0.960
Number of Major PI mutations, median (IQR)	—	0.0 (0.0–0.0)	2.0 (0.0–2.0)	**< 0.001**
Number of NRTI mutations, median (IQR)	—	0.0 (0.0–0.0)	4.0 (2.0–5.0)	**< 0.001**
Number of NNRTI mutations, median (IQR)	—	0.0 (0.0–0.0)	2.0 (1.0–3.0)	**< 0.001**
**ART regimen, n (%)**				0.524
ABC/3TC/ATV/r	12 (30.0)	11 (33.3)	18 (39.1)	
AZT/3TC/ATV/r	20 (50.0)	10 (30.3)	17 (37.0)	
TDF/3TC/ATV/r	6 (15.0)	10 (30.3)	10 (21.7)	
ABC/3TC/LPV/r	2 (5.0)	2 (6.1)	1 (2.2)	

**Abbreviations:** 3TC, lamivudine; ABC, abacavir; ART, antiretroviral therapy; ATV/r, ritonavir-boosted atazanavir; AZT, zidovudine; IQR, interquartile range; LPV/r, ritonavir-boosted lopinavir; NNRTI, non-nucleoside reverse transcriptase inhibitor; NRTI, nucleoside reverse transcriptase inhibitor; PI, protease inhibitor; TDF, tenofovir disoproxil fumarate; VF-NR, virological failure without resistance; VF-R, virological failure with resistance; VL, viral load; VS, virological suppression.

Continuous variables are presented as median (IQR) and categorical variables as *n* (%).

— Not determined because genotypic resistance testing (GRT) was not performed among participants with virological suppression (VS).

† ART duration data were unavailable for six participants (four VF-R and two VF-NR) and were excluded from duration-specific analyses.

*P* values were obtained using the Kruskal–Wallis test across the three clinical groups.

*P* values were obtained using Pearson’s χ^2^ test.

Comparison restricted to participants with VF (VF-NR versus VF-R), because virological suppression status defined the VS group.

**Table 2.** Circulating plasma miRNA expression across clinical groups with relative fold-change estimates

miRNA	VS (n = 40) ΔCq, median (IQR)	VF-NR (n = 33) ΔCq, median (IQR)	VF-R (n = 46) ΔCq, median (IQR)	Mean-based FC VF-NR vs VS	Median-based (Sensitivity Analysis) FC VF-NR vs VS	Mean-based FC VF-R vs VS	Median-based FC (Sensitivity Analysis) VF-R vs VS	Kruskal–Wallis H	P value
**miR146a-5p**	−0.06 (−0.87–1.00)	−0.74 (−1.70–0.33)	−1.75 (−4.26–−0.18)	1.86	1.60	3.77	3.23	16.61	**<0.001**
**miR150-5p**	0.38 (−1.56–1.12)	1.03 (0.30–1.83)	2.65 (0.91–3.22)	0.47	0.64	0.25	0.21	24.37	**<0.001**
**miR29a-3p**	−1.61 (−2.34–−0.21)	−0.04 (−1.54–1.89)	1.96 (−0.42–3.06)	0.39	0.34	0.16	0.08	28.86	**<0.001**

**Abbreviations:** VS, virological suppression; VF-NR, virological failure without drug resistance; VF-R, virological failure with drug resistance; FC, fold change. ΔCq values are presented as median (IQR). Mean-based fold-change estimates were calculated using the conventional 2^−ΔΔCq^ method based on group mean ΔCq values, with the VS group as the calibrator. Median-based fold-change estimates were calculated as a sensitivity analysis using group median ΔCq values. Lower ΔCq values indicate higher relative miRNA expression.

**Table 3.** Holm-adjusted pairwise comparisons of circulating plasma miRNA expression

miRNA	Comparison	Mann–Whitney U	Raw *P*	Holm-adjusted *P*	Effect size (*r*)
**miR-146a-5p**	VS vs VF-NR	850.0	**0.036**	0.071	0.246
	VS vs VF-R	1376.0	**<0.001**	**<0.001**	0.426
	VF-NR vs VF-R	958.0	**0.048**	0.071	0.223
**miR-150-5p**	VS vs VF-NR	450.0	**0.020**	**0.020**	0.272
	VS vs VF-R	390.0	**<0.001**	**<0.001**	0.495
	VF-NR vs VF-R	457.0	**0.003**	**0.005**	0.338
**miR-29a-3p**	VS vs VF-NR	403.0	**0.004**	**0.009**	0.333
	VS vs VF-R	329.5	**<0.001**	**<0.001**	0.551
	VF-NR vs VF-R	481.5	**0.006**	**0.009**	0.310

**Abbreviations:** VS, virological suppression; VF-NR, virological failure without drug resistance; VF-R, virological failure with drug resistance. Pairwise comparisons were performed only following a significant Kruskal–Wallis test. Holm’s procedure was applied within each miRNA to control the family-wise error rate across the three planned pairwise comparisons.

**Table 4.** Spearman correlations between circulating plasma miRNAs and clinical characteristics

Variable	miR-146a-5p ρ (q)	miR-150-5p ρ (q)	miR-29a-3p ρ (q)
**Overall study population (n = 119)**			
Viral load	0.252 **(0.025)**	−0.322 **(<0.001)**	−0.365 **(<0.001)**
ART duration	0.121 (0.355)	−0.227 **(0.048)**	−0.359 **(<0.001)**
PI duration	−0.006 (0.949)	−0.090 (0.489)	−0.070 (0.592)
**Virological failure subgroup (n = 79)**			
Viral load	−0.058 (0.752)	−0.029 (0.882)	−0.014 (0.947)
Major PI mutation count	0.281 **(0.042)**	−0.107 (0.489)	−0.117 (0.489)
NRTI mutation count	0.354 **(0.005)**	−0.050 (0.769)	−0.158 (0.315)
NNRTI mutation count	0.225 (0.107)	−0.214 (0.122)	−0.230 (0.107)

**Abbreviations:** ρ, Spearman’s rank correlation coefficient; q, Benjamini–Hochberg false discovery rate-adjusted *P* value; ART, antiretroviral therapy; PI, protease inhibitor; NRTI, nucleoside reverse transcriptase inhibitor; NNRTI, non-nucleoside reverse transcriptase inhibitor. Correlations with viral load, ART duration and PI duration were assessed in the overall study population. Correlations with viral load and HIV drug resistance mutation counts were assessed separately among participants with virological failure. Benjamini–Hochberg correction was applied across all reported correlation analyses. Raw *P* values are provided in **Supplementary Table 5**.

**Table 5.** Diagnostic performance of circulating plasma miRNA models for virological failure and HIV drug resistance

Comparison	Marker	Apparent AUC (95% CI)	Optimism Corrected AUC	Sensitivity	Specificity
**VF vs VS**	miR-146a-5p	0.704 (0.607–0.792)	-	40.5%	97.5%
	miR-150-5p	0.734 (0.645–0.819)	-	59.5%	85.0%
	miR-29a-3p	0.768 (0.669–0.853)	-	59.5%	87.5%
	**Combined three-miRNA panel**	**0.893 (0.829–0.949)**	**0.883**	**87.3%**	**82.5%**
**VF-R vs VF-NR**	miR-146a-5p	0.631 (0.499–0.750)	-	50.0%	78.8%
	miR-150-5p	0.699 (0.572–0.813)	-	65.2%	84.8%
	miR-29a-3p	0.683 (0.560–0.800)		69.6%	66.7%
	**Combined three-miRNA panel**	**0.769 (0.660–0.862)**	**0.740**	**52.2%**	**97.0%**

Abbreviations: AUC, area under the receiver operating characteristic curve; CI, confidence interval; VF, virological failure; VS, virological suppression; VF-NR, virological failure without drug resistance; VF-R, virological failure with drug resistance. Receiver operating characteristic (ROC) analyses were performed using logistic regression models. AUCs are presented with bootstrap-derived 95% confidence intervals (2,000 bootstrap replicates). Optimal thresholds were determined using the Youden index. Optimismcorrected AUCs are shown for the combined three-miRNA models following internal bootstrap validation. Estimated positive predictive values (PPV) and negative predictive values (NPV) for assumed virological failure prevalences of 20%, 25% and 30% are presented in Supplementary Table 6.

**Table 6.** Paired bootstrap comparison of diagnostic performance between combined three-miRNA panel and best-performing individual miRNA

Clinical comparison	Best individual miRNA	Best individual miRNA AUC	Combined three-miRNA panel AUC	ΔAUC (95% CI)	*P*-value
**VF vs VS**	miR-29a-3p	0.768	0.893	0.125 (0.061–0.218)	**<0.001**
**VF-R vs VF-NR**	miR-150-5p	0.699	0.769	0.070 (−0.015–0.259)	0.109

**Abbreviations:** AUC, Area Under the Curve; CI, Confidence Interval; miRNA, microRNA; VF, virological failure; VS, virological suppression; VF-NR, virological failure without drug resistance; VF-R, virological failure with drug resistance. Differences in diagnostic performance were evaluated using paired bootstrap resampling (2,000 replicates) with model refitting in each bootstrap sample. The observed difference in area under the receiver operating characteristic curve (ΔAUC) was calculated as the AUC of the combined three-miRNA logistic regression model minus the AUC of the best-performing individual miRNA. Bootstrap-derived 95% confidence intervals and two-sided *P*-values are reported.

**Table 7.** Multivariable logistic regression analysis of factors associated with virological failure and HIV drug resistance

Variable	VF (n = 113)			HIVDR (n = 73)		
	aOR	95% CI	*P*-value	aOR	95% CI	*P*-value
**miR-146a-5p**	3.11	1.80–5.39	**<0.001**	1.44	1.04–1.99	**0.027**
**miR-150-5p**	0.43	0.26–0.70	**0.001**	0.67	0.46–0.96	**0.031**
**miR-29a-3p**	0.77	0.56–1.06	0.110	1.12	0.81–1.55	0.490
**Age (years)**	0.97	0.93–1.02	0.259	1.04	0.99–1.08	0.109
**Sex (female)**	0.61	0.17–2.15	0.443	1.75	0.58–5.29	0.322
**ART duration (years)**	1.32	1.09–1.60	**0.005**	1.18	1.01–1.38	**0.040**
**PI duration (years)**	0.78	0.59–1.03	0.080	0.94	0.75–1.18	0.614

**Abbreviations:** aOR, adjusted odds ratio; ART, antiretroviral therapy; CI, confidence interval; HIVDR, HIV drug resistance; PI, protease inhibitor. Logistic regression analyses were performed using **−ΔCq** values, such that higher values correspond to higher miRNA expression. Multivariable models were adjusted for age, sex, ART duration and PI duration. Participants with missing ART duration data (n = 6) were excluded from the adjusted analyses.

## Data Availability

The HIV-1 protease and reverse transcriptase sequences analysed in this study form part of a larger dataset comprising 297 sequences deposited in the NCBI GenBank repository under accession numbers PX475350–PX475646, characterising HIV-1 drug resistance among adults receiving second-line protease inhibitor-based antiretroviral therapy in Zimbabwe, as previously reported. The 79 participants with virological failure included in the present miRNA biomarker analysis represent a defined subset of this deposited dataset. The GenBank accession numbers corresponding to the participants included in the present study are provided in **Supplementary Table 1**. TaqMan^™^ miRNA assay identifiers, reference gene expression stability data, Healthy Control expression data, **PPV/NPV diagnostic performance data** and the raw (unadjusted) correlation analysis outputs supporting the findings of this study are provided in **Supplementary Tables 2–6**. The participant-level clinical, virological and miRNA expression datasets analysed during the current study are available from the corresponding author (Justin Mayini, jmayini@brti.co.zw) upon reasonable request.

## Abbreviations

aOR: Adjusted Odds Ratio
ART: Antiretroviral Therapy
AUC: Area Under the Curve
BRTI: Biomedical Research and Training Institute
cDNA: Complementary Deoxyribonucleic Acid
CI: Confidence Interval
Cq: Quantification cycle
CV: Coefficient of Variation
DRM: Drug Resistance Mutation
EDTA: Ethylenediaminetetraacetic Acid
FC: Fold Change
GRT: Genotypic Resistance Testing
HC: Healthy Control
HIV: Human Immunodeficiency Virus
HIVdb: Stanford HIV Drug Resistance Database
HIVDR: HIV Drug Resistance
IQR: Interquartile Range
MCoE: Mpilo Centre of Excellence
MIQE: Minimum Information for Publication of Quantitative Real-Time PCR Experiments
miRNA: MicroRNA
MoHCC: Ministry of Health and Child Care
MRCZ: Medical Research Council of Zimbabwe
NNRTI: Non-Nucleoside Reverse Transcriptase Inhibitor
NPV: Negative Predictive Value
NRTI: Nucleoside Reverse Transcriptase Inhibitor
NTC: No-Template Control
PI: Protease Inhibitor
PLWH: People Living with HIV
PPV: Positive Predictive Value
PR: Protease Region
ROC: Receiver Operating Characteristic
RNA: Ribonucleic Acid
RT: Reverse Transcriptase
RT-qPCR: Reverse Transcription Quantitative Polymerase Chain Reaction
SD: Standard Deviation
SMCH: Sally Mugabe Central Hospital
TND: Target Not Detected
VF: Virological Failure
VF-NR: Virological Failure Without Resistance
VF-R: Virological Failure with Resistance
VL: Viral Load
VS: Virologically Suppressed
WHO: World Health Organization
